# Inhibition of glutamatergic trigeminal nucleus caudalis- vestibular nucleus projection neurons attenuates vestibular dysfunction in the chronic-NTG model of migraine

**DOI:** 10.1186/s10194-023-01607-z

**Published:** 2023-06-30

**Authors:** Yun Zhang, Yixin Zhang, Yanyun Wang, Xiaoyan Zhang, Guangcheng Qin, Dunke Zhang, Lixue Chen, Jiying Zhou

**Affiliations:** 1 Department of Neurology, The First Affiliated Hospital of Chongqing Medical University, 1st You Yi Road, Yu Zhong District, Chongqing, 400016 China; 2 Laboratory Research Center, The First Affiliated Hospital of Chongqing Medical University, Chongqing, China

**Keywords:** Migraine, Vestibular dysfunction, Glutamate, CGRP, Neural circuit

## Abstract

**Background:**

Prior clinical studies suggest a shared mechanism between vestibular symptoms and migraine headache. However, the specific neuroanatomical substrate connecting vestibular symptoms with migraine remains to be largely unknown. Thus, the aim of this study was to further investigate the mechanisms that whether and how trigeminovestibular neurons produce effects on neuronal activation in vestibular nucleus (VN).

**Methods:**

A chronic-NTG rat model was established by recurrent intermittent administration of nitroglycerin (NTG). Pain- and vestibular-related behaviors were assessed. To selectively inhibit the glutamatergic neurons and trigeminal nucleus caudalis (TNC) to VN projection neurons, the AAVs encoding engineered Gi-coupled hM4D receptor were administered in the TNC or VN area.

**Results:**

We identify a glutamatergic projection from TNC to VN that mediates vestibular dysfunction in a chronic-NTG rat model. Inhibition of the Glutamate^TNC^ neurons alleviates vestibular dysfunction in the chronic-NTG rat. Calcitonin gene-related peptide (CGRP)-expressing neurons in the VN received glutamatergic projections from TNC neurons. Silencing the glutamatergic TNC-VN projection neurons attenuates vestibular dysfunction in the chronic-NTG rat.

**Conclusions:**

Together, we reveal a modulatory role of glutamatergic TNC-VN projection neurons in vestibular dysfunction of migraine.

**Graphical Abstract:**

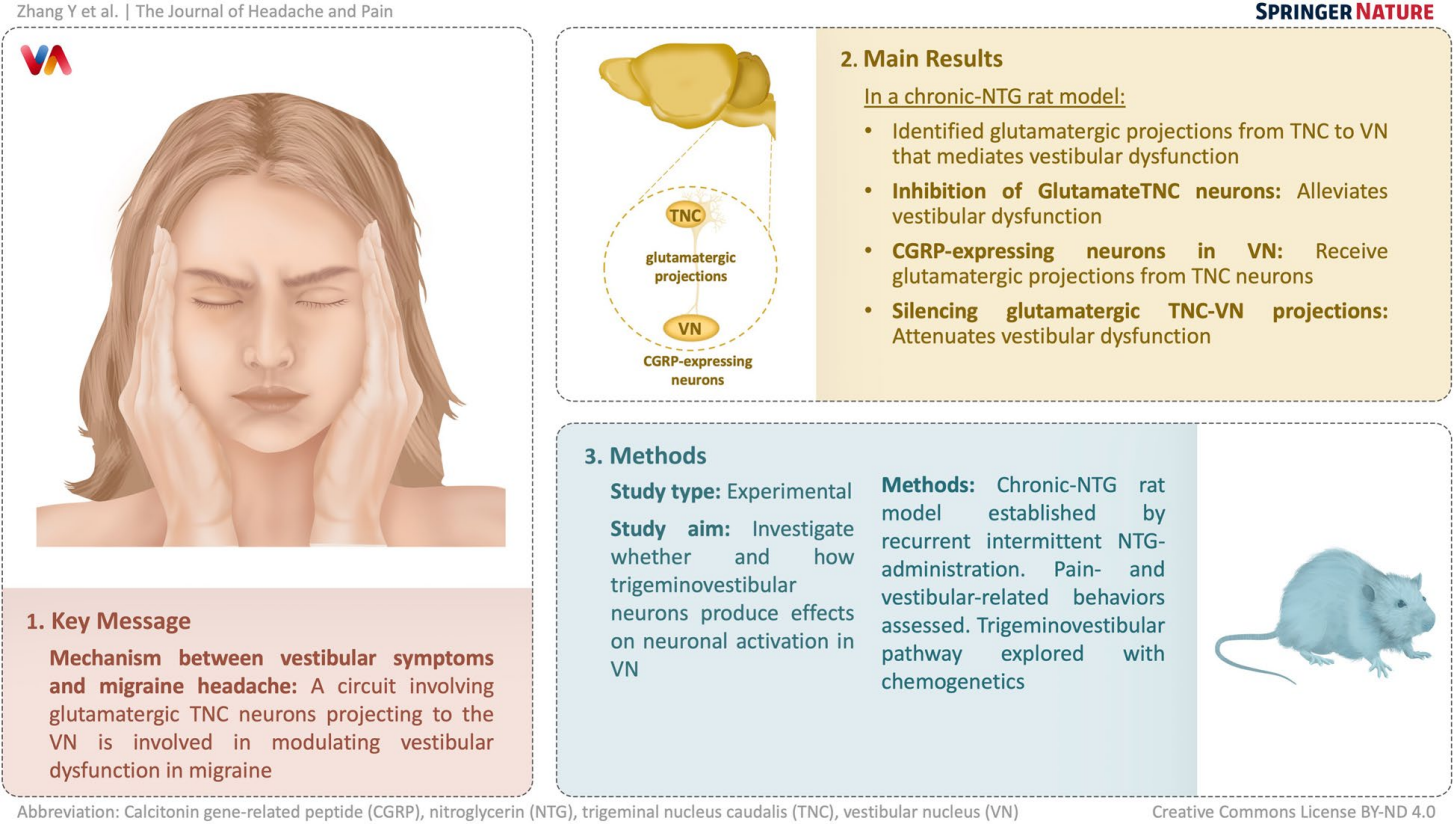

## Background

The association between migraine and vestibular symptoms is becoming increasingly recognized [1]. Lifetime prevalence study shows that 51.7% of patients with migraine experience vestibular symptoms [2]. A recent meta-analysis demonstrates that the relative frequency of headache phase-associated dizziness is 35.7%, while headache phase-associated vertigo is 33.9% among patients with migraine [1]. Vestibular suppressants are commonly prescribed symptomatically to migraine patients with vestibular symptoms, but the efficacy is limited [3]. To date, the underlying mechanisms of vestibular symptoms in migraine are largely unknown, rendering migraine with vestibular symptoms difficult to treat.

Accumulating evidence from studies regarding brain networks [4–7], neurotransmitter systems [8, 9], and neuroplasticity [10] has revealed that migraine headache and episodes of vestibular symptoms may be caused by the same processes. Neuroimaging studies show that numerous brain regions, such as the thalamus, dorsal pons, midbrain, temporal cortex, and cerebellum, are involved in the central mechanisms of vestibular symptoms in migraine [4–7]. Preclinical studies indicate that vestibular symptoms may be due to sensitization and activation of the trigeminovascular system causing release of the calcitonin gene-related peptide (CGRP). Inhibiting neuronal activities of the trigeminal nucleus caudalis (TNC) simultaneously attenuates neuronal activation of the vestibular nucleus (VN) in the chronic-nitroglycerin (NTG) rat model [9, 10]. However, how the trigeminovascular system may influence the vestibular system is still poorly understood.

Magnetic resonance spectroscopy studies find that glutamate levels in the central nociceptive pathway, such as the thalamus [11] and visual cortex [12], are significantly increased in patients with migraine compared to controls during the interictal phase. Meanwhile, local concentrations of other metabolites, like gamma-aminobutyric acid, show no statistical differences between migraine and controls in both studies [11, 12]. Prior animal studies demonstrate that glutamatergic synapses in the TNC area strengthen nociceptive transmission, primarily contributing to the central sensitization in migraine [13, 14]. Moreover, the highest proportion of trigeminovestibular neurons are glutamatergic neurons (up to 37%) [15]. These raise the possibility that the glutamatergic trigeminovestibular neurons could have an important role in processing vestibular symptoms among migraine patients.

We previously identified CCRP as a promising target for reversing vestibular dysfunction in a chronic-nitroglycerin (NTG) rat model [10]. We have shown that the expression changes of CGRP, as well as CGRP1 receptor components, calcitonin receptor-like receptor (CLR) and receptor activity modifying protein 1 (RAMP1), within the VN are associated with the NTG treatment in a time-dependent manner [10]. We also observed that chronic treatment of BIBN4096BS, a selective CGRP1 receptor antagonist, can reduce the neuronal activation in the TNC and VN, and prevent the development of acute and chronic allodynia, as well as vestibular dysfunction in a chronic-NTG rat model [10]. Considering the behavioral evidence that inhibition of activated neurons in the VN could be beneficial for the treatment of vestibular symptoms in migraine, the aim of this study was to further investigate the mechanisms that whether and how glutamatergic trigeminovestibular neurons produce effects on neuronal activation in the VN.

## Methods

### Animals

All the animal procedures and ethics of the experiments in this study were approved by the Institutional Animal Care and Use Committee of Chongqing Medical University (Chongqing, China) (2022-K98). All animal experiments complied with the ARRIVE guidelines.

Rats were purchased from the Experimental Animal Center of Chongqing Medical University (Chongqing, China). Male Sprague–Dawley (SD) rats weighing approximately 220 g were randomly assigned to behavioral, immunofluorescence staining and neuronal tracing studies, while male and female SD rats weighing approximately 120 g were equally distributed in the whole-cell patch-clamp experiment to ensure neuronal activity. All animals were reared under a 12 h light–dark cycle at a controlled temperature (23 ± 1℃) and were fed food and water ad libitum.

### Chronic-NTG rat model

Chronic-NTG rat model was established as previously described [10]. In brief, stock solution was prepared as follows: 5 mg/ml NTG (Beijing Regent, China) was dissolved in 30% alcohol, 30% propylene glycol and water. Before each injection, the stock solution was diluted to 1 mg/ml with 0.9% saline. All rats were randomly received intraperitoneal injection of 10 mg/kg of diluted NTG stock solution or 0.9% saline at an equal volume every other day for 9 days (5 times in total). After the fifth injection, rats were then placed back to the cages until sacrifice.

### Stereotaxic surgery procedures

As described previously, rats were anesthetized with sodium pentobarbital (50 mg/kg, intraperitoneal injection) [9]. When the pedal reflex was absent, they were placed on a stereotaxic apparatus (ST-51603; Stoelting Co, Chicago, IL, USA) with a heating pad. After shaving and cleaning the operation region with iodine and medical alcohol, the scalp was incised to expose the skull. The tissue covering the skull was bluntly dissected with cotton swabs. The precise coordinates of the TNC and the VN were determined using the rat brain atlas of Paxinos and Watson (6^th^ edition) [16]. The following coordinates of the TNC (Interaural: AP = -5.40 mm; ML = -3 mm; DV = -8.5 mm), and the VN (Interaural: AP = -1.20 mm; ML = -1.60 mm; DV = -7.5 mm) were applied for stereotaxic surgery. Craniotomy holes (1 mm diameter) were drilled followed by drug infusion with microliter syringes (1 μl,10 μl, Gaoge, Shanghai). Keeping the microliter syringes vertically, a volume of 1 μl per spot was slowly injected over 3 min. After injection, the syringe was left in place for an additional 10 min and then slowly retracted. After surgery, the region was cleaned with iodine and medical alcohol, and the incision was sewn with surgical sutures. Ratswere housed separately after operation and were fed water containing antibiotics daily for 1 week.

### Experimental design and animal groups

As described in Table 1, this study contained 3 experiments:

**Table 1:** Experimental design and animal groups

	Experiment 1	Experiment 2	Experiment 3
Aims	To characterize the cell organization of trigeminal nucleus caudalis (TNC) -vestibular nucleus (VN) pathway	To determine the role of glutamatergic trigeminal neurons in migraine-related vestibular dysfunction	To determine whether the Glu^TNC^-CGRP^VN^ pathway are involved in migraine-related vestibular dysfunction
Subjects	Wild-type rats	chronic-nitroglycerin (NTG) rats	chronic-NTG rats
Interventions	1. Retrograde tracer Fluoro-Gold (FG) was injected in the VN area; Anterograde tracer virus H129-G4 was injected into the TNC area	1. AAV-Ca2 + /calmodulin-dependent protein kinase IIα (CaMKIIα, an enzyme in glutamatergic neurons)-hM4D-mCherry virus was injected in the TNC area; 2. Intraperitoneal injection of CNO or saline	1. AAV-EF1α-DIO-hM4D-mCherry virus was injected in the TNC area, while AAV_retro_-Syn-Cre was simultaneously injected in the VN area; 2. Intraperitoneal injection of CNO or saline
Groups	1. FG group (*n* = 5); 2. H129-G4 group (*n* = 5)	1. Saline group (*n* = 6); 2. Chronic-NTG group (*n* = 6); 3. Chronic-NTG + CNO group (*n* = 6); 4. Chronic-NTG + AAV-CaMKIIα-hM4D-mCherry + saline (*n* = 6); 5. Chronic-NTG + AAV-CaMKIIα-hM4D-mCherry + CNO group (*n* = 6)	1. Saline group (*n* = 6); 2. Chronic-NTG group (*n* = 6); 3. Chronic-NTG + CNO group (*n* = 6); 4. Chronic-NTG + AAV-EF1α-DIO-hM4D-mCherry + saline (*n* = 6); 5. Chronic-NTG + AAV-EF1α-DIO-hM4D-mCherry + CNO group (*n* = 6)
Observations	1. TNC area: colocalization of FG + and glutamate/ GABA + neurons; 2. VN area: colocalization of H129-G4 + and calcitonin gene-related peptide (CGRP) CGRP + neurons	1. Behavior studies; 2. Colocalization of glutamate and AAV-CaMKIIα-hM4D-mCherry in the TNC; 3. C-fos + neurons in VN area; 4. Detection of miniature excitatory postsynaptic currents (mEPSCs) of TNC-projecting VN neurons	1. Behavior studies

- *Experiment 1* To characterize the cell organization of TNC—VN pathway, rats were randomly divided into two groups to perform administration of retrograde tracer (Fluoro-Gold, FG) or anterograde tracer virus (H129-G4).
- *Experiment 2* To determine the role of glutamatergic trigeminal neurons in migraine-related vestibular dysfunction, rats were randomly divided into five groups with or without the administration of AAV-Ca2 + /calmodulin-dependent protein kinase IIα (CaMKIIα, an enzyme in glutamatergic neurons)-hM4D-mCherry and Clozapine-N-oxide (CNO) (*n* = 6/group).
- *Experiment 3* To determine whether the Glu^TNC^-CGRP^VN^ pathway are involved in migraine-related vestibular dysfunction, rats were randomly divided into five groups with or without the administration of AAV-EF1α-DIO-hM4D-mCherry combined with AAV_retro_-Syn-Cre virus and CNO (*n* = 6/group).

### Drug administration

The retrograde tracer Fluoro-Gold (FG,2% solution dissolved in saline, Fluorochrome LLC, USA) and anterograde tracer virus H129-G4 (1.0*10^9^ PFU/ml, GeneChem, Shanghai, China) were used in this study. FG was unilaterally injected into the VN area. The tissues were examined 2 weeks after FG injection. H129-G4 was unilaterally injected into the TNC area. The tissues were examined 1.5 days after H129-G4 injection because of viral characteristics. To selectively label glutamatergic neurons in the TNC area, the AAV encoding engineered Gi-coupled hM4D receptor (AAV-Ca2 + /calmodulin-dependent protein kinase IIα (CaMKIIα)-hM4D-mCherry, in the unilateral TNC area, 1.15 × 10^13^ v.g./ml, 1 μl, OBiO Technology Co.Ltd., China) was administered 2 weeks before the serial NTG injections (Fig. 2). To chemogenetically inhibit activities of glutamatergic neurons, CNO (selectively activate AAV-containing receptor, 3 mg/kg, Sigma-Aldrich, USA) was administered intraperitoneally after the serial NTG injections (Fig. 2). To selectively label TNC-VN projection neurons, the AAV-EF1α-DIO-hM4D-mCherry (in the unilateral TNC area, 7.37 × 10^12^ v.g./ml, 1 μl, OBiO Technology Co.Ltd., China) and AAVretro-Syn-Cre (in the unilateral VN area, 1.77 × 10^12^ v.g./ml, 1 μl, OBiO Technology Co.Ltd., China) were administered 2 weeks before the serial NTG injections (Fig. 5). To chemogenetically inhibit activities of TNC-VN projection neurons, CNO (3 mg/kg, Sigma-Aldrich, USA) was administered intraperitoneally after the serial NTG injections (Fig. 5). The control group was intraperitoneally administered saline. The injection sites were all on the right side.

### Behavioral tests

All behavioral tests were performed by two experimenters under double-blind conditions as described previously [9]. In this study, all the basal responses, including periorbital and hind paw mechanical pain and hind paw thermal pain threshold, were assessed 0.5 h prior to each NTG/saline administration. All post-treatment responses, including mechanical pain threshold and thermal pain threshold, as well as balance beam walk and negative geotaxis tests, were evaluated 2 h after each NTG/saline administration. To compare the behavioral changes between the viral-inducers CNO or saline pain- and vestibular—related behavioral tests were additionally evaluated at 0.5 h after CNO/saline injection. Rats were adaptively trained before the first vestibular—related behavioral tests.

#### Assessment of mechanical allodynia

Mechanical allodynia tests were performed with a von Frey instrument (Aesthesio) as previously described [14]. Briefly, starting with a small force value which was gradually increased, the von Frey hairs were applied perpendicularly to the periorbital region or the central area of the hind paw surface The cut-off was 100 g. A positive response was confirmed when the rats’ head or paw exhibited a withdrawal response. The values corresponding to the positive response were considered as one threshold. The interval period was more than 5 min between applications until three positive responses occurred. Then, the average threshold of three positive-response thresholds was calculated.

#### Assessment of thermal hyperalgesia

Thermal hyperalgesia was determined using a Hargreaves radiant heat apparatus (model PL-200, IITC, Taimeng, Chengdu, China) as previously described [9, 10]. Rats were placed above the equipment in a transparent cage, and allowed to adapt for approximately 30 min until the animals no longer exhibited exploratory behaviors before the formal experiment. Then, infrared radiation (intensity:50%) was aligned with the central area of rat hind paws. The values of withdrawal latency were automatically in recorded on the display screen once the rat experienced a sudden paw-withdrawal response. The maximum allowable duration of thermal stimulation was 20 s in case the rats were injured. The interval period was more than 5 min between applications for three trials per rat. Then, the average withdrawal latency of the three trial values was calculated.

#### Balance beam walk

The protocol of the balance beam walk was described in a previous study [9, 10]. In brief, a balance beam (length: 190 cm, diameter: 2.5 cm) was placed horizontally 40 cm above the ground. A cushion was placed under the beam to prevent the rats from falling. The duration was the time that the rats crossed the entire balance beam. The maximum allowable duration of the experiment was 90 s for rest. The interval period was more than 5 min between applications for three trials per rat. The data were discarded when rats fell or failed to pass. Then, the average duration of the three trials was calculated. When the average was more than 60 s it was recorded as 60 s.

#### Negative geotaxis

The protocol of negative geotaxis was performed as previously described [9, 10]. Rats were placed on a 40° slope with their head downward, and the duration for a turn of 180° upward was recorded. The maximum allowable duration of the experiment was 90 s for rest. The interval period was more than 5 min between applications for three trials per rat. The data were discarded when the rats failed to turnback. Then, the average duration of the three trials was calculated. When the average was more than 60 s it was recorded as 60 s.

### Immunofluorescence staining

Rats were anesthetized with sodium pentobarbital (50 mg/kg, intraperitoneal injection,). The rats were intracardially perfused with 0.1 M PBS 250 ml, followed by 250 ml of 4% paraformaldehyde (PFA). Brain tissues were postfixed in 4% PFA overnight at 4 ℃, and cryoprotected at 4 ℃ by successively immersing them in gradually increasing concentration of sucrose solution (20% to 30%) until the tissues sank. The tissue was then frozen at -80 ℃ until subsequent experiments. Transverse sections containing the VN and TNC area were cut at 16 μm on a cryostat (Leica, Japan). The coordinates of the TNC and VN were determined according to the rat brain atlas of Paxinos and Watson (6^th^ edition). Antigen recovery was performed by treating the sections with sodium citrate buffer and heating them in a microwave for 15 min. Sections were permeabilized with 0.3% Triton X-100 for 10 min at 37 ℃, and then blocked in 10% normal goat serum (Boster, China) for 30 min at 37 ℃. Then, sections were incubated at 4 ℃ overnight with the following primary antibodies: anti-glutamate (1:400, G6642, Sigma, USA), anti-GABA (1:400, A2052, Sigma, USA), anti-c-Fos (1:5000, NBP2-50,057, Novus Biologicals, USA), anti-CGRP (1:100, sc-57053, Santa Cruz, USA). The brain sections were rinsed with 3 times (5 min each) in PBS, followed by a 90 min incubation in the dark at 37 ℃ with the following secondary antibodies: DyLight 488-conjugated goat anti-rabbit IgG (1:400, A23220, Abbkine, China); DyLight 594-conjugated goat anti-rabbit IgG (1:400, A23420, Abbkine, China); Cy3-conjugated goat anti-mouse IgG (1:400, Beyotime, China), and then washed again with PBS for 3 times (10 min each). The nuclei were stained with DAPI. The sections were then cover-slipped with 50% glycerol. Images were acquired with a fluorescence confocal microscope (ZEISS, Germany).

For post hoc immunofluorescence, sections were immediately obtained after whole-cell patch clamp recording, in which the patched cells were labeled with neuronbictin-488, and were immersed in 4% PFA for post-fixation at 4 ℃ for 1 week. Sections were rinsed with 0.01 M 3 times (5 min each) in PBS, permeabilized with 0.3% Triton X-100 for 10 min at 37 ℃, and then blocked in 10% normal goat serum (Boster, China) for 30 min at 37 ℃. Sections were incubated at overnight at 4 ℃ with an anti-CGRP (1:100, sc-57053, Santa Cruz, USA) antibody. After incubation sections were rinsed with 3 times (5 min each) with PBS, followed by a 90 min incubation in the dark at 37 ℃ with Cy3-conjugated goat anti-mouse IgG (1:400, Beyotime, China) secondary antibodies and then washed again 3 times (10 min each) with PBS. Finally, the sections were cover-slipped with 50% glycerol covering. Images were acquired with a fluorescence confocal microscope (ZEISS, Germany).

As described previously, morphological identifications of TNC and VN were under microscope [9, 10]. AAV-CaMKIIα-hM4D-mCherry + , AAV-EF1α-DIO-hM4D-mCherry + , C-Fos + , CGRP + , FG + , Glutamate + , GABA + , and H129-G4 + were quantified on ipsilateral side for TNC and VN from selected serial transverse sections. Sections were collected from the rostral to caudal part. Each section was separated by at least 250 μm to avoid repeated counting [9]. Through the optical fractionator method, the number of target cells was assessed under × 200 magnification (*n* = 5 or 6 rats per group, 5 images per rat).

### Slice preparations

Coronal brainstem slices, containing medial vestibular nucleus (MVN), were collected from rats as described previously [10]. Briefly, rats were decapitated after deep anesthesia with 1% (50 mg/kg, intraperitoneal injection) sodium pentobarbital. Brains were rapidly dissected from the skull and transferred to ice-cold cutting solution [composition (in mM): 60 mM NaCl, 100 mM surose, 2.5 mM KCl, 1.25 mM NaH_2_PO_4_, 20 mM D-glucose, 26 mM NaHCO_3_, 1 mM CaCl_2_, 5 mM MgCl_2_]. Coronal brainstem slices were cut at 300-μm thickness using a vibratome (Leica VT1200S) after approximately 1 min of freezing. Then, the slices were placed on a supporting net and submerged in pre-warmed (32℃) and oxygenated artificial cerebrospinal fluid (ACSF) [composition (in mM): 125 mM NaCl, 3 mM KCl, 1.25 mM NaH_2_PO_4_, 15 mM D-glucose, 26 mM NaHCO_3_, 2 mM CaCl_2_, 2 mM MgCl_2_] for at least 50 min before recording at room temperature (24–26℃). All the solutions were continuously saturated with 95% O_2_ and 5% CO_2._

### Whole-cell patch-clamp recording

Patch electrodes were pulled from borosilicate glass (ITEM #:BF150-86–10, Sutter Instrument Co.) using a vertical micropipette puller (PC-100, NARISHIGE, JAPAN). The resistance of the microelectrodes was 4–6 MΩ when filled with internal solution. In this study, the excitatory-specific pipette internal solution contained 130 mM CsCH_3_SO_3_, 10 mM HEPES, 10 mM CsCl, 4 mM NaCl, 1 mM MgCl_2_, 1 mM EGTA, 5 mM NMG, 5 mM MgATP, 0.5 mM Na_2_ GTP, and 12 mM phosphocreatine (pH adjusted to 7.2 with CsOH, 275–290 mOsm). Electrophysiological recordings were acquired using a patch-clamp amplifier (Multiclamp 700B, Molecular Devices) in voltage-clamp mode filtered at 2 kHz and digitized at 10 kHz ( Digidata 1440A and PClamp10.3, Molecular Devices). The recordings were performed using ACSF at a recording chamber perfusion rate of 2 mL/min. The miniature excitatory post-synaptic currents (mEPSCs) were recorded in the presence of 1 μM tetrodotoxin (TTX, CAS No.:4368–28-9, Chengdu Must Bio-Technology Co.,Ltd), 0.1 mM picrotoxin (PTX, CAS: 124–87-8, APEXBIO) and strychnine hydrochloride (CAS:1421–86-9,MACKLIN). Voltage-clamp recordings were performed at a holding potential of -70 mV. The cells were visualized using infrared-differential interference contrast microscopy (Olympus, Tokyo, Japan), and the microelectrodes were positioned using a micromanipulator. After a tight seal (electrode resistance > 1 GΩ) was formed between the electrode tip and the cell surface, slight suction was briefly applied until a whole-cell patch-clamp recording configuration was achieved (access resistance < 20 MΩ). The series resistance (15–30 MΩ) was compensated automatically using a MultiClamp 700B and was monitored throughout the recording, and the data were discarded if the resistance changed by more than 20%. The data were analyzed using Mini Analysis (Synaptososoft, Leonia, NJ, USA) and Clampfit 10.3. All the other chemicals used in this electrophysiological experiment were purchased from Sigma-Aldrich (St. Louis, MO, USA).

### Statistical analysis

The data in this study are presented as the mean ± standard error deviation (mean ± SD). Parametric analysis was analyzed by using paired *t*-test, while nonparametric analysis was analyzed by using Mann–Whitney *U* test. One or Two-way ANOVA with Bonferroni post hoc analysis was used for statistical comparisons among groups. Cumulative probability plots were generated with the Kolmogorov–Smirnov test. SPSS 22.0 software (SPSS Inc., IBM, USA) and GraphPad Prism version 8.0 (GraphPad Software Inc., CA, USA) were used for statistical analysis and graph generation. *P* < 0.05 indicates a significant difference.

The rats used for behavioral statistical analysis were verified by histological to ensure the location of viral transduction. The data were deleted for analysis if viral transduction extended beyond the target regions.

## Results

### Dissection of the TNC-VN circuit

**Fig. 1:**
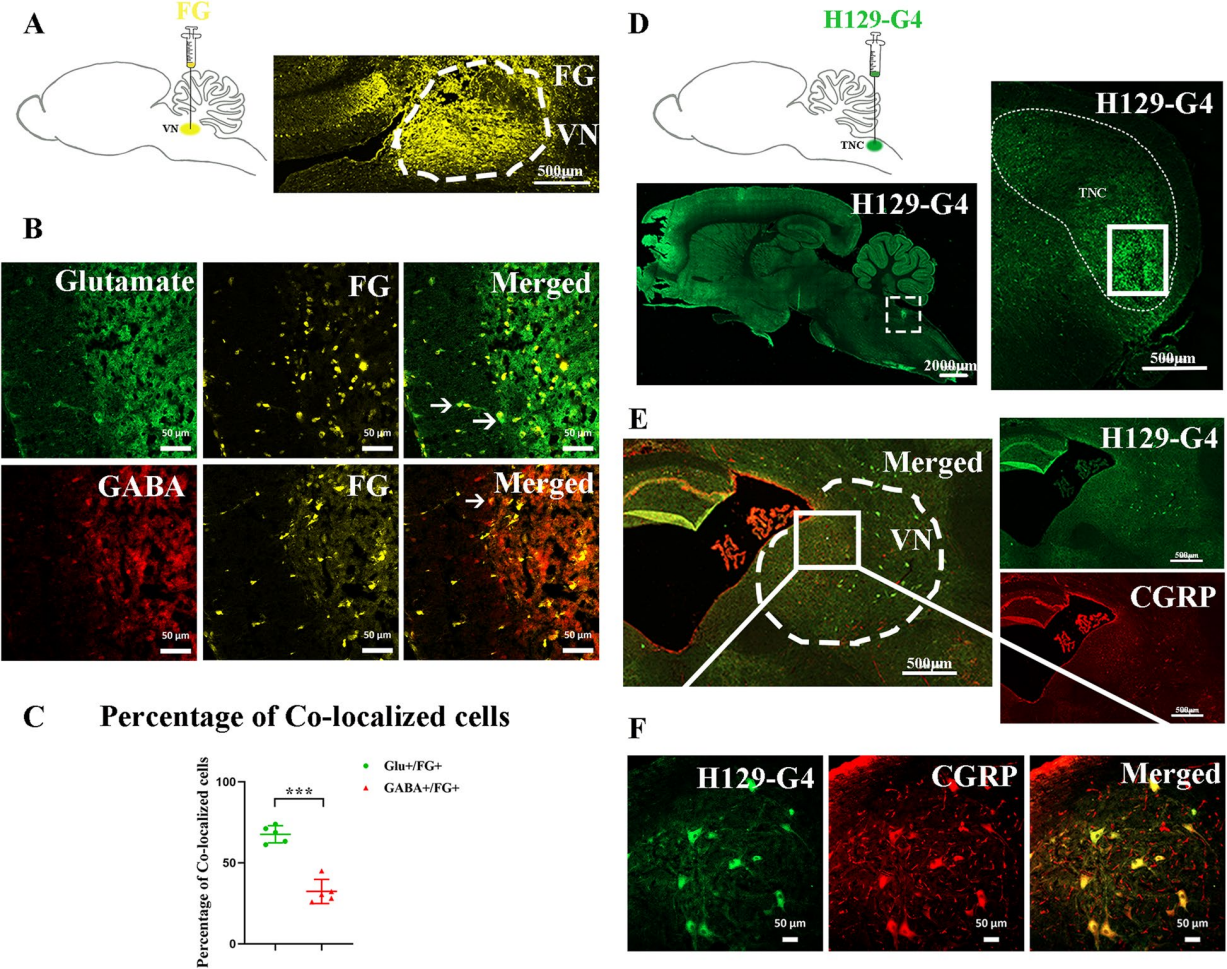
Dissection of Glutamate^TNC^-CGRP^VN^ circuit. **A** (Left) Schematic showing the site of FG injection into the unilateral VN of WT rat. (Right) Representative image of FG injection site in VN at transverse plane. White dashed line circle showing VN region. Scale bar, 500 μm. **B** Representative images showing the co-localization of Glutamate (upper row, green) or GABA (lower row, red) and FG (gold) in TNC. Arrowhead indicates co-localized neurons. Scale bar, 50 μm. **C** Percentage of glutamate + /FG + neurons among glutamate + neurons (present as green dot), and GABA + /FG + neurons among GABA + neurons (present as red triangle) in TNC. *N* = 5 rats/group, each dot represents the average data of one rat. Mann–Whitney *U* test, ***p < 0.0001. **D** (Left upper row) Schematic showing the site of H129-G4 injection into the unilateral TNC of WT rat. (Left lower row) Representative image of H129-G4 injection site in TNC at sagittal plane. White dashed box showing the injection site. Scale bar, 2000 μm. (Right) Representative image of H129-G4 injection site in TNC at transverse plane. White dashed line circle showing TNC region. White box showing the injection site. Scale bar, 500 μm. **E** Representative images showing the co-localization of H129-G4 (green) and CGRP (red) in VN. White dashed line circle showing VN region. Scale bar, 500 μm. **F** Magnified view of **E** image. Scale bar, 50 μm

First, the FG, used as a retrograde tracer, was stereotaxically delivered into the unilateral VN of wild-type rat (Fig. 1A). Two weeks later, FG-labeled neurons were identified in the superficial lamina of ipsilateral TNC, which were predominantly co-localized with the glutamate antibody (Glutamate vs. GABA: 67.8 ± 5.3% vs. 32.3 ± 7.5%, *p* = 0.008, Fig. 1B, C). These results suggest that Glutamate^TNC^ neurons can project to VN region.

To further identify the cell type connections between TNC and VN neurons, the H129-G4 virus, an anterograde polysynaptic tracer, was applied to identify the type of VN neurons receiving TNC projections. After 1.5 days, we observed H129-G4 + cell bodies in VN region, and 71.4 ± 20.7% of cells were co-localized with the CGRP antibody based on immunofluorescence staining (Fig. 1D-F).

### Glutamate^TNC^ neurons mediate chronic-NTG induced pain behaviors and vestibular dysfunction

**Fig. 2:**
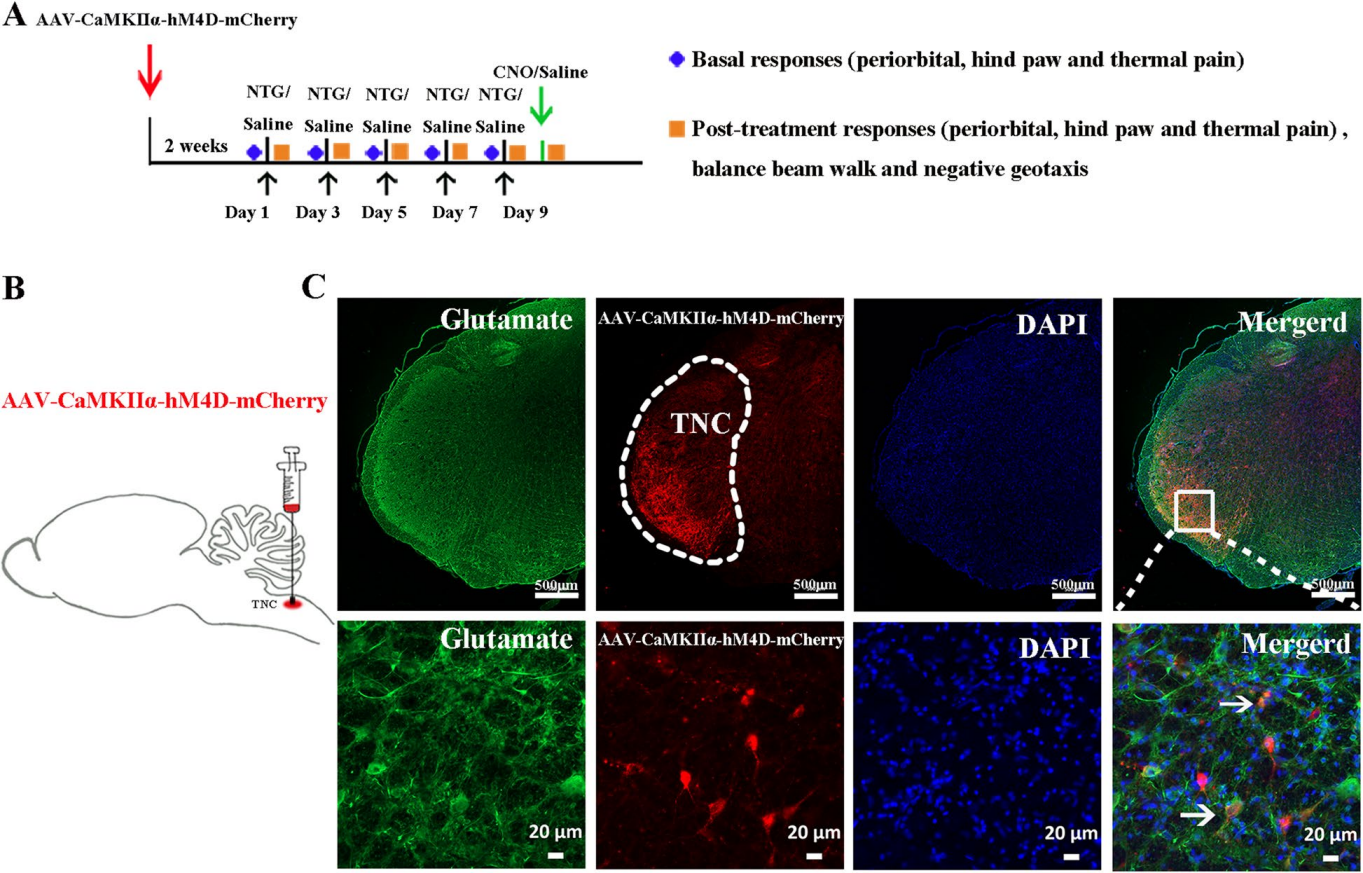
Interventions on glutamatergic neurons in chronic-NTG treated rat model. **A** Schematic illustration of intervention protocol. **B** Schematic showing the site of AAV-CaMKIIα-hM4D-mCherry injection into the unilateral TNC of chronic-NTG treated rat. **C** (Upper row) Representative images showing the co-localization of AAV-CaMKIIα-hM4D-mCherry (red) and glutamate (green) in TNC. White dashed line circle showing TNC region. Scale bar, 500 μm. (Lower row) Magnified view of image at upper row. Arrowhead indicates co-labeled neurons. Scale bar, 20 μm

We then examined the functions of TNC-VN excitatory projection. AAV-CaMKIIα-hM4D-mCherry was unilaterally injected into the TNC (Fig. 2A, B). Only glutamatergic neurons were labeled by mCherry flag (Fig. 2C, D). The average efficacy of virus transfection in Glutamate^TNC^ neurons was 71.1%.

**Fig. 3:**
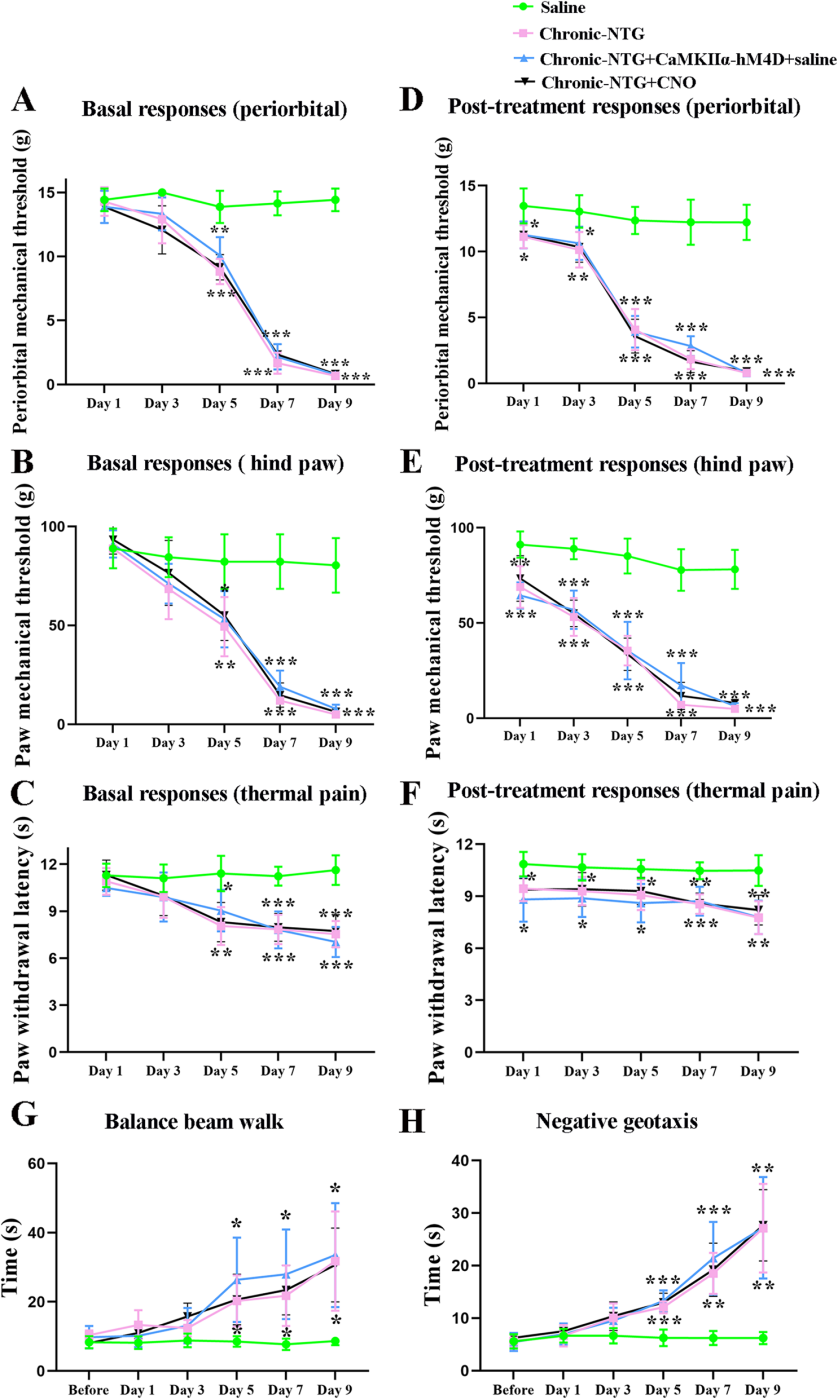
Inhibition of glutamatergic TNC neurons alleviated mechanical allodynia and thermal hyperalgesia in chronic-NTG treated rats. Basal responses of periorbital (**A**) and hind paw (**B**) mechanical, and thermal (**C**) thresholds among three groups. Post-treatment responses of periorbital (**D**) and hind paw (**E**) mechanical, and thermal (**F**) thresholds among three groups. Balance beam walk (**G**) and negative geotaxis test (**H**) among three groups. *N* = 6 rats/group. Two-way ANOVA with the Bonferroni post hoc test, *p < 0.05 compared with saline, **p < 0.005 compared with saline, ***p < 0.001 compared with saline

Chronic-NTG, chronic-NTG + CNO, and chronic-NTG + AAV-CaMKIIα-hM4D-mCherry + saline treated rats had significantly lower thresholds for mechanical stimulation (Fig. 3A, B), and shorter latencies to noxious heat (Fig. 3C) on day 5, 7 and 9 (Fig. 2A) when compared with saline group. Each NTG administration could cause marked acute hypersensitivity (Fig. 3D-F). In line with our previous studies [9, 10], chronic injection of NTG significantly extended the time in balance beam (Fig. 3G) and negative geotaxis test (Fig. 3H) on day 5, 7 and 9 when compared with the saline group. No statistically differences in pain- and vestibular- related behaviors were detected between chronic-NTG and chronic-NTG + AAV-CaMKIIα-hM4D-mCherry + saline group.

**Fig. 4:**
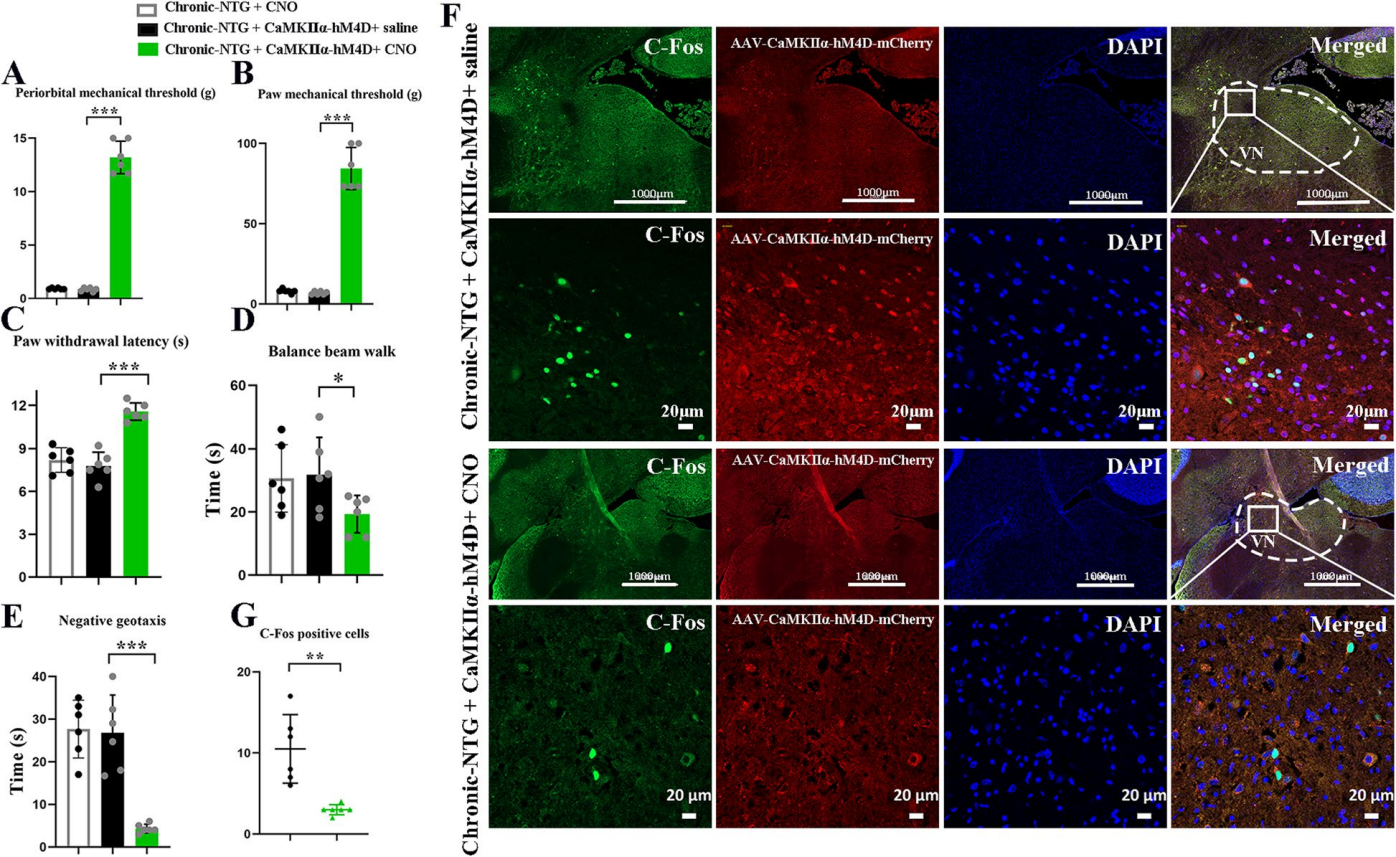
Silencing of GlutamateTNC neurons diminished chronic NTG-induced pain- and vestibular- related behaviors, as well as neuronal activation in VN. Post-treatment responses of periorbital (**A**) and hind paw (**B**) mechanical, and thermal (**C**) thresholds among three groups. Balance beam walk (**D**) and negative geotaxis test (**E**) among three groups. *N* = 6 rats/group, each dot represents the average data of one rat. Two-way ANOVA with the Bonferroni post hoc test, *p < 0.05 compared with chronic-NTG + CNO group, **p < 0.005 compared with chronic-NTG + CNO group, ***p < 0.001 compared with chronic-NTG + CNO group. **F** Representative images showing the co-localization of AAV-CaMKIIα-hM4D-mCherry (red) and C-Fos (green) in VN. The second row in each group showing the magnified view of the first row. Scale bar, 1000 μm, 20 μm

Silencing of Glutamate^TNC^ neurons with CNO diminished chronic NTG-induced pain- and vestibular- related behaviors (Fig. 4A-E). Meanwhile, the behavioral outcomes were similar between chronic-NTG + CNO group and chronic-NTG + AAV-CaMKIIα-hM4D-mCherry + saline group. Compared to chronic-NTG + AAV-CaMKIIα-hM4D-mCherry + saline group, the expression of C-Fos + in the VN neurons was significantly decreased in chronic-NTG + AAV-CaMKIIα-hM4D-mCherry + CNO group (CNO vs. saline: 3.0 ± 0.6 vs. 10.5 ± 4.2, *p* = 0.002, Fig. 4F, G).

### TNC-VN circuit modulates chronic-NTG induced pain behaviors and vestibular dysfunction

**Fig. 5:**
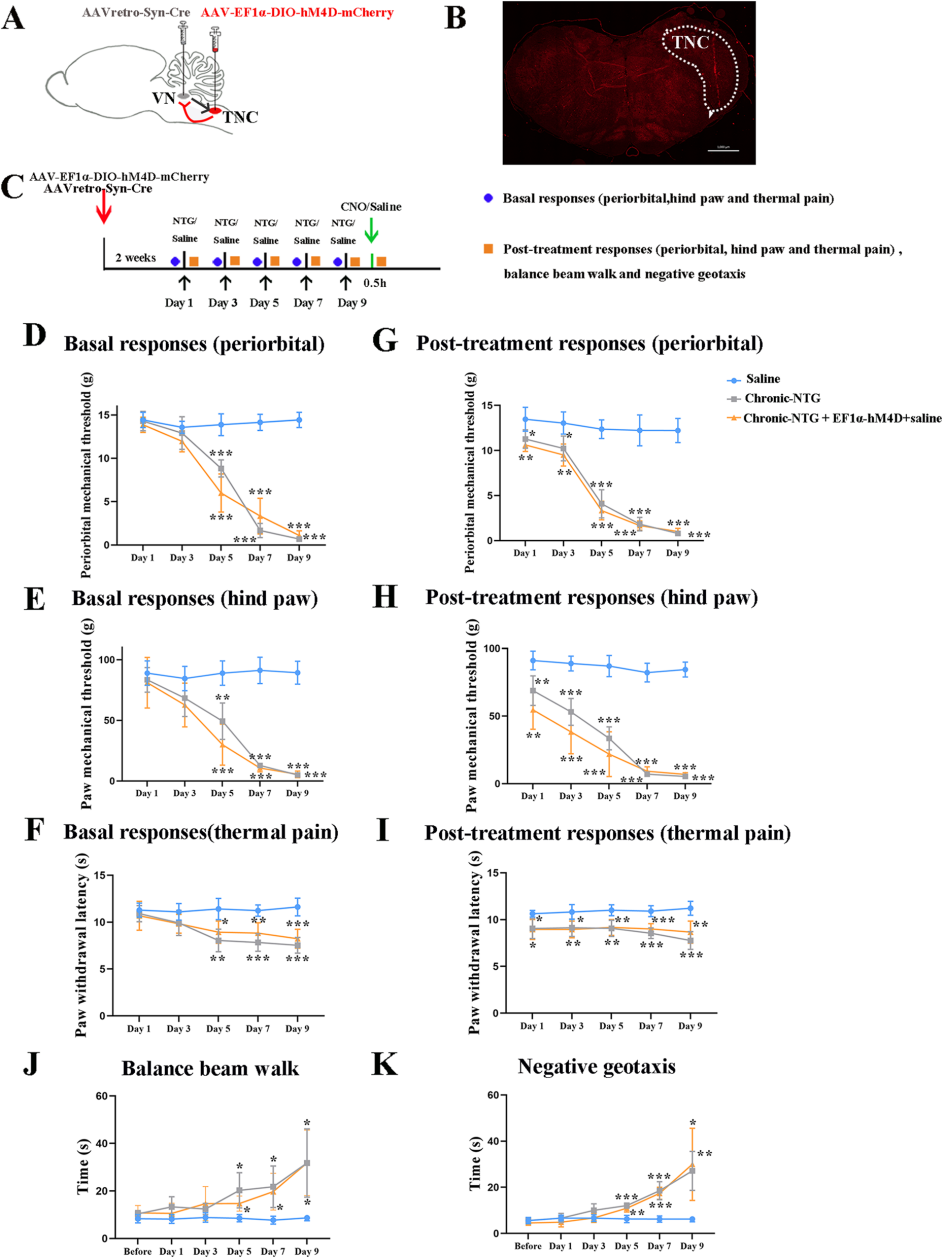
Interventions on TNC-VN circuit in chronic-NTG treated rat model. **A** Schematic showing the site of AAV-EF1α-DIO-hM4D-mCherry injection into the unilateral TNC, and the site of AAV_retro_-Syn-Cre injection into the VN at the same site of chronic-NTG treated rat. **B** Expression of AAV-EF1α-DIO-hM4D-mCherry (red) in trigeminovestibular neurons. White dashed line circle showing the TNC region. Scale bar, 1000 μm. **C** Schematic illustration of intervention protocol. Basal responses of periorbital (**D**) and hind paw (**E**) mechanical, and thermal (**F**) thresholds among three groups. Post-treatment responses of periorbital (**G**) and hind paw (**H**) mechanical, and thermal (**I**) thresholds among three groups. Balance beam walk (**J**) and negative geotaxis test (**K**) among three groups. *N* = 6 rats/group. Two-way ANOVA with the Bonferroni post hoc test, *p < 0.05 compared with saline, **p < 0.005 compared with saline, ***p < 0.001 compared with saline

**Fig. 6:**
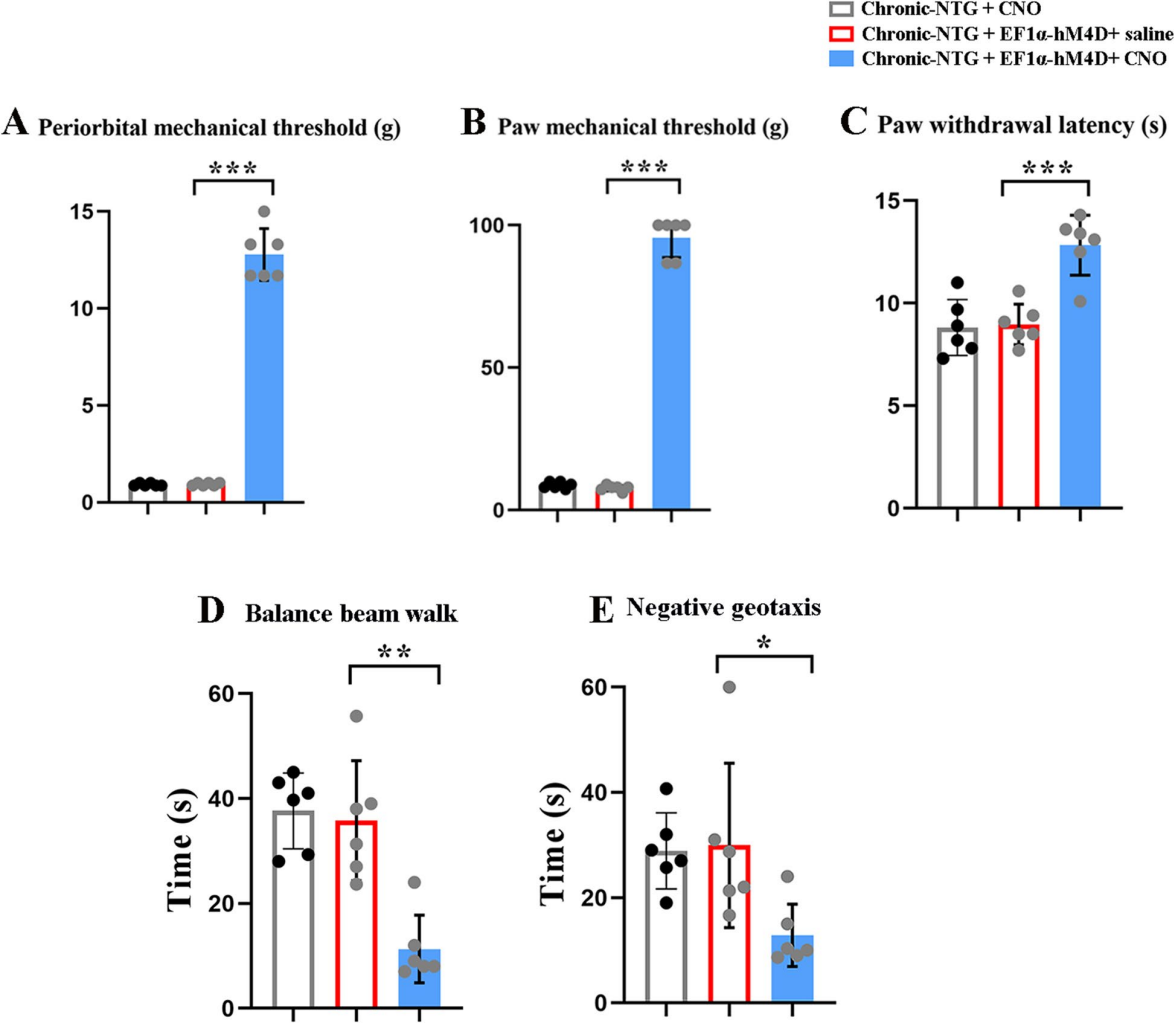
Pharmacogenetic suppression of TNC-VN circuit attenuated mechanical allodynia, thermal hyperalgesia and vestibular dysfunction in chronic-NTG treated rats. Post-treatment responses of periorbital (**A**) and hind paw (**B**) mechanical, and thermal (**C**) thresholds among three groups. Balance beam walk (**D**) and negative geotaxis test (**E**) among three groups. *N* = 6 rats/group, each dot represents the average data of one rat. Two-way ANOVA with the Bonferroni post hoc test, *p < 0.05 compared with chronic-NTG + CNO group, **p < 0.005 compared with chronic-NTG + CNO group, ***p < 0.001 compared with chronic-NTG + CNO group

To determine the role of TNC-VN circuit in the chronic-NTG model, the AAV-EF1α-DIO-hM4D-mCherry virus and AAV_retro_-Syn-Cre was simultaneously infused (Fig. 5A-C). Pain- and vestibular- related behaviors showed similar results between chronic-NTG and chronic-NTG + AAV-EF1α-DIO-hM4D-mCherry + saline group (Fig. 5D-K). Pharmacogenetic suppression of TNC-VN circuit with CNO significantly increased mechanical and thermal pain thresholds when compared with chronic-NTG + CNO group and chronic-NTG + AAV-EF1α-DIO-hM4D-mCherry + saline group (Fig. 6A-C). Inhibition of TNC-VN circuit significantly attenuated NTG-induced vestibular dysfunction, as evidenced by less time in traversing the balance beam and negative geotaxis test compared to chronic-NTG + AAV-EF1α-DIO-hM4D-mCherry + saline group (Fig. 6D, E). No significant differences were detected between chronic-NTG + CNO group and chronic-NTG + AAV-EF1α-DIO-hM4D-mCherry + saline group.

### Presynaptic mechanism maybe involved in the TNC-VN circuit of chronic-NTG treated rats

**Fig. 7:**
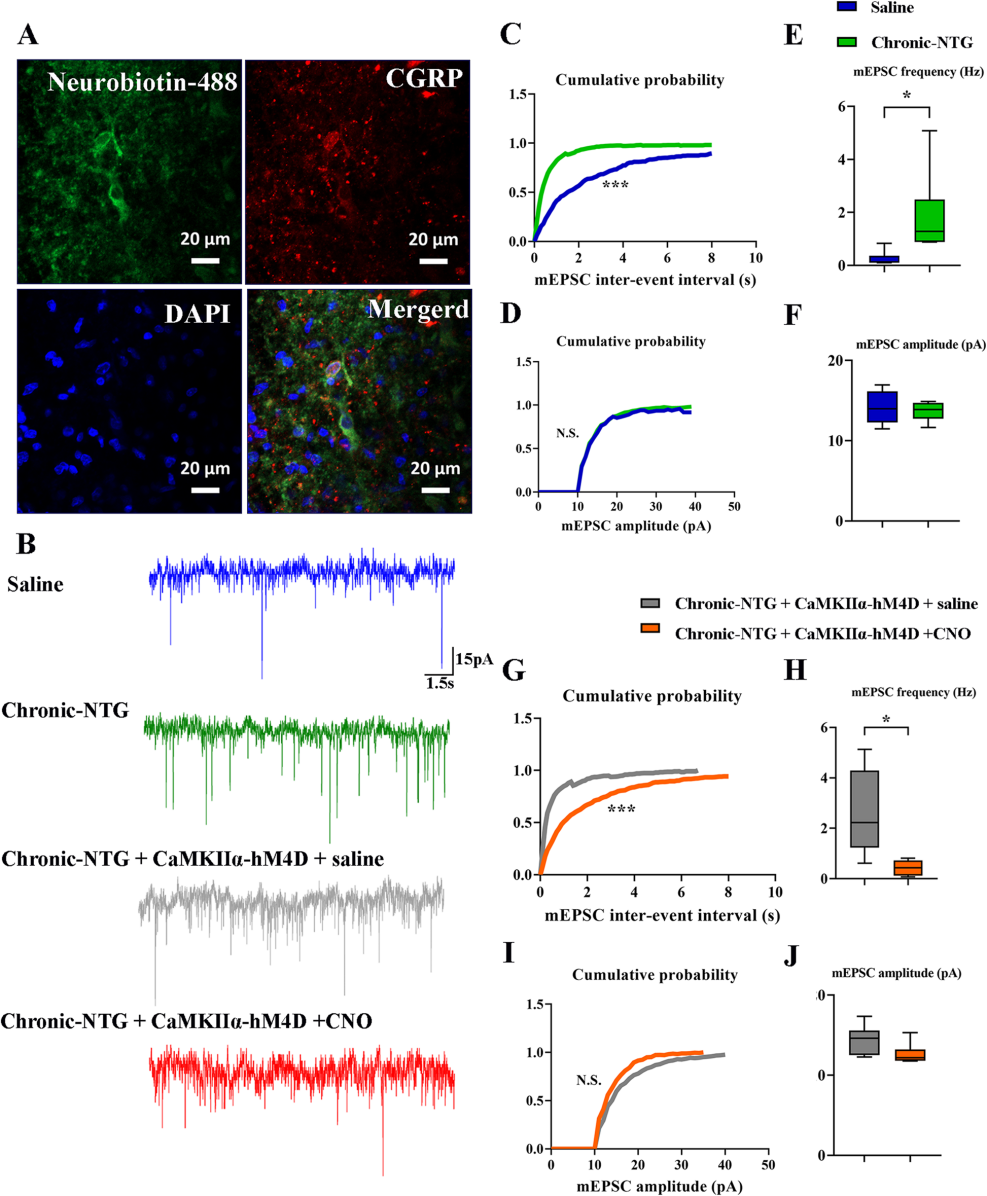
Presynaptic mechanism maybe involved in the TNC-VN circuit of chronic-NTG treated rat. **A** Representative images of the recording CGRP + neurons (neurobiotin-488: green; CGRP: red) by post hoc staining after patch. Scale bar, 20 μm. **B** Representative traces of the mEPSCs recorded in the CGRP + VN neurons in saline, chronic-NTG, chronic-NTG + AAV-CaMKIIα-hM4D-mCherry + saline, and chronic-NTG + AAV-CaMKIIα-hM4D-mCherry + CNO group. Cumulative fraction of inter-event interval (**C**) and amplitude (**D**) of the mEPSCs in the saline (blue line) and chronic-NTG (green line) group. Statistic results of the frequency (**E**) and amplitude (**F**) of mEPSCs in the saline (blue box) and chronic-NTG (green box) group. Cumulative fraction of inter-event interval (**G**) and amplitude (**H**) of the mEPSCs in the saline (blue line) and chronic-NTG (green line) group. Statistic results of the frequency (**I**) and amplitude (**J**) of mEPSCs in the saline (blue box) and chronic-NTG (green box) group (*n* = 6 neurons from 6 rats). Paired *t*-test. ***p < 0.001

In the absence of stimuli, miniature synaptic transmission results from neurotransmitter release [17]. To further determine the modulation of Glutamate^TNC^ neurons on CGRP^VN^ neurons, we recorded miniature excitatory post-synaptic currents (mEPSCs) in the VN with the presence of TTX, PTX, and strychnine hydrochloride. The frequencies of mEPSCs were significantly increased in chronic-NTG group compared to saline group (NTG vs. saline: 1.8 ± 1.6 Hz vs. 0.3 ± 0.3 Hz, *p* = 0.04, Fig. 7B, E). Moreover, a cumulative fraction plot showed a significant decrease of inter-event-interval in chronic-NTG group (Fig. 7C). As to the amplitude of mEPSCs, no evident changes were found (NTG vs. saline: 13.7 ± 1.2 pA vs. 14.1 ± 2.0 pA, *p* = 0.6, Fig. 7D, F). After chemogenetic inhibition of Glutamate^TNC^ neurons, the frequencies of mEPSCs were observed to have a significant decrease (CNO vs. saline: 0.4 ± 0.3 Hz vs. 2.6 ± 1.7 Hz, *p* = 0.01, Fig. 7B, I), while inter-event-interval was significantly increased in chronic-NTG + AAV-CaMKIIα-hM4D-mCherry + CNO group (Fig. 7G). No significant difference was detected in the amplitude of mEPSCs between chronic-NTG + AAV-CaMKIIα-hM4D-mCherry + CNO group and chronic-NTG + AAV-CaMKIIα-hM4D-mCherry + saline group (CNO vs. saline: 12.6 ± 1.3 pA vs. 14.4 ± 1.8 pA, *p* = 0.09, Fig. 7H, J). Post hoc immunostaining showed that the recorded neurons labeled with neuronbiotin-488 in pipette solution were CGRP positive (Fig. 7A). These results implied that chronic-NTG administration enhanced excitatory synaptic transmission from Glutamate^TNC^ neurons to CGRP^VN^ neurons via increasing the probability of presynaptic neurotransmitter release in the VN.

Additionally, we didn’t detect the sex differences between males and females regarding to the frequencies (NTG group: males vs. females was 1.0 ± 0.1 Hz vs. 2.7 ± 2.1 Hz, *p* = 0.1; saline group: males vs. females was 0.4 ± 0.4 Hz vs. 0.1 ± 0.1 Hz, *p* = 0.4) and amplitude of mEPSCs (NTG group: males vs. females was 13.1 ± 1.5 pA vs. 14.2 ± 0.6 pA, *p* = 0.4; saline group: males vs. females was 13.7 ± 2.9 pA vs. 14.6 ± 1.1 pA, *p* = 0.7) between chronic-NTG group compared to saline group. Similar results were found between chronic-NTG + AAV-CaMKIIα-hM4D-mCherry + CNO group and chronic-NTG + AAV-CaMKIIα-hM4D-mCherry + saline group as the frequencies (CNO group: males vs. females was 0.6 ± 0.4 Hz vs. 0.3 ± 2.0 Hz, *p* = 0.4; saline group: males vs. females was 2.8 ± 1.0 Hz vs. 2.4 ± 2.4 Hz, *p* = 0.7) and amplitude of mEPSCs (CNO group: males vs. females was 12.2 ± 0.4 pA vs. 13.1 ± 1.9 pA, *p* = 1.0; saline group: males vs. females was 14.8 ± 2.3 pA vs. 14.0 ± 1.5 pA, *p* = 1.0).

## Discussion

**Fig. 8:**
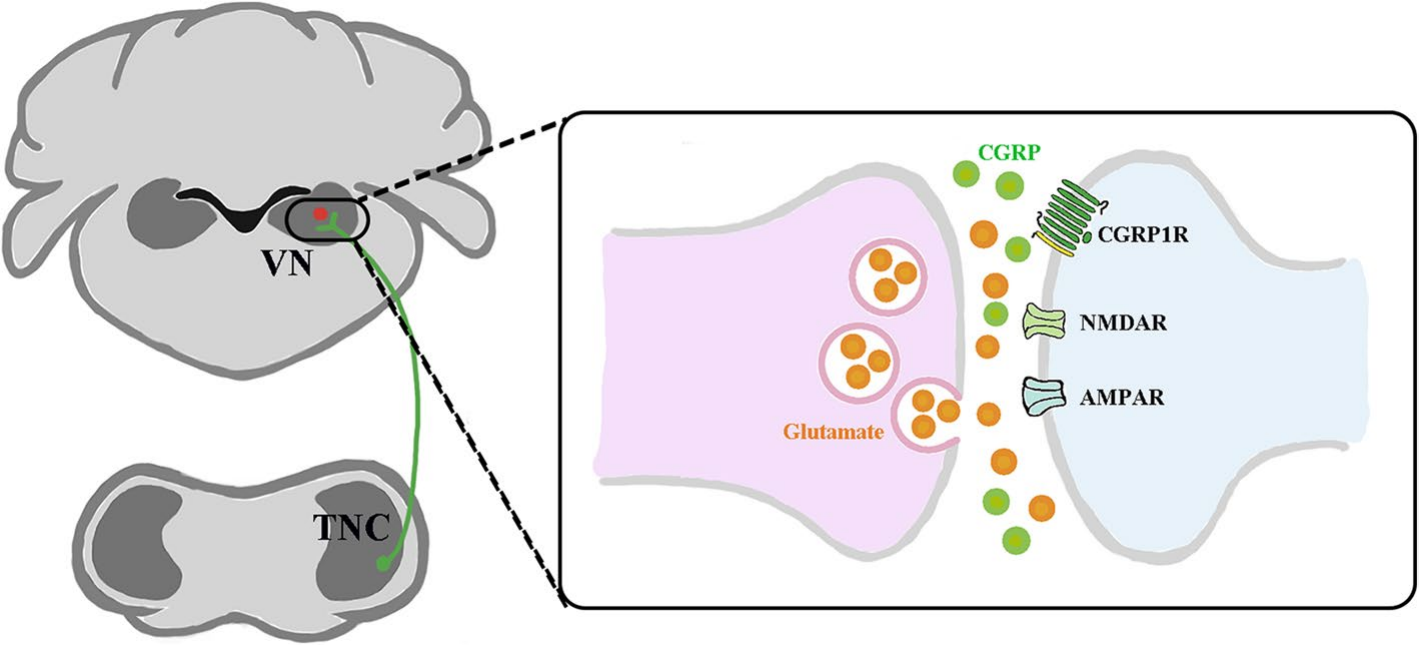
Schematic mechanism of Glutamate^TNC^ neurons projecting to CGRP^VN^ neurons underlying migraine-related vestibular dysfunction

This study defines a glutamatergic TNC–VN circuit, which is involved in vestibular dysfunction under chronic-NTG administration in a rat model. Central to this process is a circuit mechanism involving the enhanced synaptic transmission of Glutamate^TNC^ neurons projecting to CGRP.^VN^ neurons (Fig. 8). It is important to note that reducing the activity of this circuit alleviates the nociception and vestibular dysfunction. These findings suggest that suppressing the activity of this circuit may play a role in coping with vestibular dysfunction in chronic-NTG treated rat model.

The relationship between migraine headache and vestibular symptoms has been shown to be quite complex. Vestibular symptoms have been reported not only during premonitory and headache phase, but also interictal phase of migraine episodes [1, 18, 19]. Human brain imaging studies have shown a similar alternations of gray matter volume, and rhythmic activity of the vestibulo-thalamo-cortical pathway between migraine patients with and without vestibular symptoms [7, 19]. Prior clinical studies show that vestibular abnormalities could be also detected during the symptom-free interval in migraine patients with and without vestibular symptoms [19–22]. These findings indicate a shared mechanism between vestibular dysfunction and migraine headache. The central integration of nociception system and vestibular pathways at cortex and thalamus level has been frequently reported in human studies [7, 19, 23]. However, due to current technical limitations, metabolic activities in brainstem nuclei remain to be largely unknown.

TNC is one of the essential structures modulating trigeminovascular nociceptive transmission in the development of migraine [24]. Neuroanatomic studies determined the extensive projections between TNC and VN [9, 15]. Our previous study showed that inhibiting neuronal activation in TNC via silencing synthesis of CGRP in the trigeminal ganglion, could significantly suppress activation of VN neurons in chronic-NTG treated rats [9]. Furthermore, the glutamatergic neurons projecting to VN are primarily located at spinal trigeminal nucleus orails (Sp5O), spinal trigeminal nucleus interpolaris (Sp5I) and spinal trigeminal nucleus candalis (Sp5C or TNC), while the GABAergic neurons projecting to VN are mainly located in Sp5O and a small amount of Sp5I under physiological condition [15]. In this study, we found that specifically suppressing activities of glutamatergic trigeminovestibular neurons could remarkably reduce the neuronal activation in VN, which in turn attenuates vestibular dysfunction. The current findings suggest a specific neuroanatomical substrate that underlie vestibular dysfunction in migraine.

Traditional vestibular suppressants or triptans seem to have limited effects on migraine with vestibular symptoms [3, 25]. Understanding why the treatment for migraine patients with vestibular symptoms is far from satisfactory represents a major challenge. Currently, monoclonal antibodies or antagonists targeting the CGRP pathway are recommended for migraine prevention as they are effective [26–28]. Whether migraine patients with vestibular symptoms will be beneficial to the medications targeting CGRP pathway still needs to be investigated. Immunohistochemistry staining shows a strong expression of CGRP in VN under physiological condition [29]. The expression of CGRP and its receptor components on VN neurons were significantly increased after chronic-NTG administration. Antagonizing CGRP receptor could reverse the chronic-NTG induced neuronal activation in VN and vestibular dysfunction [10]. This study showed that Glutamate^TNC^ neurons had connections with CGRP^VN^ neurons, and might influence the activities of CGRP^VN^ neurons via presynaptic mechanism. Nevertheless, other neurotransmitters, such as serotonin and GABA, are also reported in VN [30–32]. It is also demonstrated that 5-HT_1F_ receptor is co-localized with CGRP in VN [29]. Thus, the interactions between CGRP and other neurotransmitters within VN needs to be further determined.

Migraine with and without vestibular symptoms are more prevalent in women than in men [22]. Due to the higher basal level of corticosterone, females are more pain-sensitive, and greater variability in hormone responses to noxious stress compared with males [33, 34]. However, prior study fails to find behavioral differences between male and female rodents, partly due to the different menstrual cycle in rodents, but also the target candidate may have limited relevance to sexual dimorphism [35]. Additionally, vestibular symptoms have been reported among patients who have endogenous changes in the concentration of estrogen and progesterone in the premenstrual syndrome, or using exogenous hormones (such as oral contraceptives) [36]. Therefore, this study primarily chose male rats to establish the model, but the sexual dimorphism on Glutamate^TNC^-CGRP^VN^ circuit requires further exploration.

Although we emphasized the crucial role of glutamatergic trigeminovestibular neurons, other subpopulations in TNC and VN may also play roles in vestibular dysfunction after chronic-NTG administration. The AAV-based retrograde strategy could not label all the Glutamate^TNC^ neurons; thus, we cannot exclude the possibility that the unlabeled glutamatergic neurons or other neuron subpopulations in TNC project to the VN and play a role in vestibular dysfunction after chronic-NTG administration. It is also possible that the recorded CGRP + neurons in VN might receive other projections. Additionally, PPR (paired-pulse ratio) is another index representing the release probability from presynaptic terminals. Given the limitation of the details about the projection scope of the TNC-VN pathway in the VN slices, we failed to record the PPR in the present study. 

## Conclusions

In summary, our results identified a functional role for Glutamate^TNC^-CGRP^VN^ neurons in mediating migraine-related vestibular dysfunction. Inhibition of this projection might be a potential therapeutic method for the treatment of migraine-related vestibular symptoms.

## Data Availability

The data used and analyzed in this study are available from the corresponding author on reasonable request.

## Abbreviations

ACSF: Artificial cerebrospinal fluid
ANOVA: Analysis of variance
ARRIVE: Animal Research Reporting of In Vivo Experiments
CGRP: Calcitonin Gene-related Peptide
CM: Chronic migraine
CNO: Clozapine N-oxide
DAPI: 4,6-Diamidino-2-phenylindole
FG: Fluorogold
GABA: Gamma-aminobutyric acid
Glu: Glutamate
mEPSCs: Miniature excitatory postsynaptic currents
NMDA: Glutamate N-methyl-D-aspartate
NTG: Nitroglycerin
PBS: Phosphate buffered saline
PFA: Paraformaldehyde
PTX: Picrotoxin
Sp5O: Trigeminal nucleus orails
Sp5I: Trigeminal nucleus interpolaris
Sp5C: Trigeminal nucleus candalis
TNC: Trigeminal Nucleus Caudalis
TTX: Tetrodotoxin
VN: Vestibular Nucleus

## References

[1] Iljazi A, Ashina H, Lipton RB (2020). Dizziness and vertigo during the prodromal phase and headache phase of migraine: a systematic review and meta-analysis. Cephalalgia.

[2] Vukovic V, Plavec D, Galinovic I (2007). Prevalence of vertigo, dizziness, and migrainous vertigo in patients with migraine. Headache.

[3] Vuralli D, Yildirim F, Akcali DT (2018). Visual and postural motion-evoked dizziness symptoms are predominant in vestibular migraine patients. Pain Med.

[4] Borsook D, Burstein R (2012). The enigma of the dorsolateral pons as a migraine generator. Cephalalgia.

[5] Shin JH, Kim YK, Kim HJ (2014). Altered brain metabolism in vestibular migraine: comparison of interictal and ictal findings. Cephalalgia.

[6] Obermann M, Wurthmann S, Steinberg BS (2014). Central vestibular system modulation in vestibular migraine. Cephalalgia.

[7] Zhang X, Zhou J, Guo M (2023). A systematic review and meta-analysis of voxel-based morphometric studies of migraine. J Neurol.

[8] Espinosa-Sanchez JM, Lopez-Escamez JA (2015). New insights into pathophysiology of vestibular migraine. Front Neurol.

[9] Zhang Y, Zhang Y, Tian K (2020). Calcitonin gene-related peptide facilitates sensitization of the vestibular nucleus in a rat model of chronic migraine. J Headache Pain.

[10] Tian R, Zhang Y, Pan Q (2022). Calcitonin gene-related peptide receptor antagonist BIBN4096BS regulates synaptic transmission in the vestibular nucleus and improves vestibular function via PKC/ERK/CREB pathway in an experimental chronic migraine rat model. J Headache Pain.

[11] Bathel A, Schweizer L, Stude P (2018). Increased thalamic glutamate/glutamine levels in migraineurs. J Headache Pain.

[12] Zielman R, Wijnen JP, Webb A (2017). Cortical glutamate in migraine. Brain.

[13] Latremoliere A, Woolf CJ (2009). Central sensitization: a generator of pain hypersensitivity by central neural plasticity. J Pain.

[14] Zhang W, Lei M, Wen Q (2022). Dopamine receptor D2 regulates GLUA1-containing AMPA receptor trafficking and central sensitization through the PI3K signaling pathway in a male rat model of chronic migraine. J Headache Pain.

[15] Diagne M, Valla J, Delfini C (2006). Trigeminovestibular and trigeminospinal pathways in rats: retrograde tracing compared with glutamic acid decarboxylase and glutamate immunohistochemistry. J Comp Neurol.

[16] Paxinos G, Watson C (2009) The rat brain in stereotaxic coordinates. Compact sixth edition ed. Rat Brain in Stereotaxic Coordinates 3.2(6) 10.1016/0165-0270(80)90021-7 6110810

[17] Liu Y, Chen QY, Lee JH (2020). Cortical potentiation induced by calcitonin gene-related peptide (CGRP) in the insular cortex of adult mice. Mol Brain.

[18] Carvalho GF, Vianna-Bell FH, Florencio LL (2019). Presence of vestibular symptoms and related disability in migraine with and without aura and chronic migraine. Cephalalgia.

[19] Wang X, Yin Z, Lian Y (2021). Premonitory symptoms in migraine from China: a multi-clinic study of 4821 patients. Cephalalgia.

[20] Boldingh MI, Ljostad U, Mygland A (2013). Comparison of interictal vestibular function in vestibular migraine vs migraine without vertigo. Headache.

[21] Dieterich M, Obermann M, Celebisoy N (2016). Vestibular migraine: the most frequent entity of episodic vertigo. J Neurol.

[22] Radtke A, von Brevern M, Neuhauser H (2012). Vestibular migraine: long-term follow-up of clinical symptoms and vestibulo-cochlear findings. Neurology.

[23] Furman JM, Marcus DA, Balaban CD (2013). Vestibular migraine: clinical aspects and pathophysiology. Lancet Neurol.

[24] Goadsby PJ, Holland PR, Martins-Oliveira M (2017). Pathophysiology of migraine: a disorder of sensory processing. Physiol Rev.

[25] Webster KE, Dor A, Galbraith K (2023). Pharmacological interventions for acute attacks of vestibular migraine. Cochrane Database Syst Rev.

[26] Sacco S, Amin FM, Ashina M (2022). European Headache Federation guideline on the use of monoclonal antibodies targeting the calcitonin gene related peptide pathway for migraine prevention - 2022 update. J Headache Pain.

[27] Akerman S, Romero-Reyes M (2020). Preclinical studies investigating the neural mechanisms involved in the co-morbidity of migraine and temporomandibular disorders: the role of CGRP. Br J Pharmacol.

[28] Cumberbatch MJWD, Mason GS, Hill RG, Hargreaves RJ (1999). Dural vasodilation causes a sensitization of rat caudal trigeminal neurones in vivo that is blocked by a 5-HT1B/1D agonist. Br J Pharmacol.

[29] Ahna SK, Khalmuratovaa R, Jeona SY, Kima JP, Parka JJ, Hur DG, Balaban CD (2009). Colocalization of 5-HT1F receptor and calcitonin gene-related peptide in rat vestibular nuclei. Neurosci Lett.

[30] Halberstadt AL, Balaban CD (2006). Serotonergic and nonserotonergic neurons in the dorsal raphe nucleus send collateralized projections to both the vestibular nuclei and the central amygdaloid nucleus. Neuroscience.

[31] Ahn SK, Khalmuratova R, Jeon SY (2009). Colocalization of 5-HT1F receptor and glutamate in neurons of the vestibular nuclei in rats. NeuroReport.

[32] Halberstadt AL, Balaban CD (2007). Selective anterograde tracing of the individual serotonergic and nonserotonergic components of the dorsal raphe nucleus projection to the vestibular nuclei. Neuroscience.

[33] Bulls HW, Freeman EL, Anderson AJ (2015). Sex differences in experimental measures of pain sensitivity and endogenous pain inhibition. J Pain Res.

[34] Zimmer CBH, Vedder H, Lautenbacher S (2003). Sex differences in cortisol response to noxious stress. Clin J Pain.

[35] Chou TM, Lee ZF, Wang SJ (2022). CGRP-dependent sensitization of PKC-delta positive neurons in central amygdala mediates chronic migraine. J Headache Pain.

[36] Rybak LP (1995). Metabolic disorders of the vestibular system. Otolaryngol Head Neck Surg.

